# Load-carrying biomechanics in children: programed exercise enhances postural resilience and reduces plantar pressure under backpack load

**DOI:** 10.3389/fpubh.2026.1887693

**Published:** 2026-08-11

**Authors:** Aida Bendo, Denis Nuriu

**Affiliations:** 1 Department of Movement and Health, Faculty of Physical Activity and Recreation, Sports University of Tirana, Tirana, Albania; 2 Department of Physical Activity and Health, Institute of Scientific Research in Sport, Sports University of Tirana, Tirana, Albania

**Keywords:** backpack load, load carriage, plantar pressure, postural resilience, schoolchildren, structured exercise

## Abstract

**Introduction:**

Daily schoolbag carriage represents a common mechanical load in school-aged children and has been associated with alterations in gait mechanics, postural control, and plantar pressure distribution. However, evidence regarding the effectiveness of structured exercise programs in reducing these biomechanical alterations during schoolbag carriage remains limited. Therefore, identifying effective intervention strategies is important for promoting musculoskeletal health during growth.

**Objective:**

To evaluate the effects of a 16-week structured exercise program on maximum plantar pressure and weight-bearing symmetry during walking with a schoolbag in school-aged children.

**Research methodology:**

A controlled non-randomized experimental study was conducted involving 147 school-aged children (experimental: *n* = 74; control: *n* = 73). Pre- and post-intervention assessments were performed using the T&T Medilogic^®^ plantar pressure system during walking with a standardized schoolbag load. Maximum plantar pressure and weight-bearing symmetry were analyzed using repeated-measures ANOVA, paired-, and independent-samples *t*-tests, with effect sizes estimated using Cohen’s *d*.

**Results:**

The experimental group demonstrated a significant reduction in maximum plantar pressure during walking with a schoolbag (*p* = 0.010; *d* = −0.31), whereas no significant change was observed in the control group (*p* = 0.37). Between-group comparisons confirmed a significant intervention effect (*p* = 0.016). The reduction in plantar pressure was accompanied by improved weight-bearing symmetry, indicating a more favorable distribution of plantar loads during functional load carriage.

**Conclusion:**

The structured exercise program reduced maximum plantar pressure and improved weight-bearing symmetry during walking with a schoolbag, supporting better postural control under functional loading conditions. These findings support the implementation of targeted school-based exercise programs to promote musculoskeletal health and reduce the biomechanical demands associated with daily schoolbag carriage in children.

## 1 Introduction

School-aged children regularly carry loads, commonly in the form of schoolbags. External loads are considered a normal part of daily activities, but carrying a load affects the musculoskeletal system in the form of modified gait, postural alignment, and distribution of plantar loads. Physically, the addition of an external load changes the center of mass position, necessitating compensatory adaptations in trunk flexion, lower limb kinematics, and muscle excitation to help maintain balance and forward propulsion. Such changes may amplify the ground reaction forces and plantar pressure, with increased heel and forefoot pressure especially affecting the effective functioning of the movement system and posture. Gait is an elaborate, dynamically controlled activity whose function requires the cooperation of neuromuscular control, sensory feedback, and biomechanical posture. It has been previously demonstrated that locomotor performance develops progressively in childhood through the development of the central and peripheral nervous systems and structural change in the musculoskeletal system (1). The development of coordinated and efficient gait patterns is closely associated with the refinement of motor control and the integration of proprioceptive, visual, and vestibular inputs, allowing for the regulation of balance and movement in different environments (2, 3). However, the addition of extra loads challenges these control mechanisms, frequently resulting in altered gait mechanics and decreased postural efficiency.

Previous research has suggested that promoting more physical activity and targeted exercise interventions can ultimately lead to improved neuromuscular coordination, muscular strength, and postural control in children. Organized programs of physical education, in particular those that address areas of balance, core stability, and functional patterns of movement, are important for enhancing gait symmetry (4, 5). These modifications are linked with increased shock absorption, improved alignment among body segments, and diminished mechanical load localized to the plantar surface. Conversely, under-exercise and sedentary behavior are related to decreased muscular capacity and a reduction of postural control, which could contribute to the biomechanical burden of external loading (6). Epidemiological data shows that there is a large prevalence among school-age children of postural deviations and gait alterations, ranging from 30 to 65% (7). These problems are further exacerbated by extrinsic influences, including excessive schoolbag weight, load distribution, and prolonged sedentary behaviors. Daily school activities requiring children to carry heavy loads can increase mechanical stress to the musculoskeletal system, leading to fatigue, discomfort, and compensatory movement strategies in response to this strain. Such adaptations, over time, will impair postural stability and predispose individuals to musculoskeletal disorders unless corrected via prevention efforts.

Several school-based intervention studies incorporating core stabilization, balance training, functional movement exercises, and lower-limb strengthening have demonstrated improvements in postural control, dynamic balance, movement coordination, lower-limb function, and overall musculoskeletal performance in school-aged children (4, 5, 8, 9). However, relatively few studies have specifically examined whether these interventions also modify plantar pressure distribution during functional tasks involving schoolbag carriage. This limitation provided the rationale for developing the present 16-week structured exercise program.

In that respect, plantar pressure analysis has become a useful and objective technique of estimating the biomechanical influence of loading. If the forces around the plantar areas are studied and their magnitude and distribution are quantified, local pressure peaks, asymmetries, and load transfer inefficiencies might be noted that would otherwise be undetectable by observation alone (10, 11). The use of such a study in a pediatric population enables a more accurate characterization of the impact of external loads on gait mechanics and postural control in practice.

Alterations in plantar pressure distribution reflect changes in the way body weight is transferred through the feet when standing and walking. Excessive or asymmetrical plantar loading may indicate compensatory movement strategies and impaired weight-bearing symmetry, potentially challenging postural control during functional activities. Consequently, interventions capable of improving plantar load distribution may also enhance weight-bearing symmetry and postural control during functional load carriage (11, 12).

Despite the growing body of evidence describing the biomechanical consequences of schoolbag carriage, most previous investigations have been limited to cross-sectional or observational designs that primarily quantify plantar pressure, gait alterations, or postural adaptations under external loading. Evidence regarding the effectiveness of structured exercise interventions aimed at modifying these biomechanical responses remains scarce, particularly in school-aged children (13, 14). Consequently, it is still unclear whether targeted exercise programs can reduce plantar loading while simultaneously improving functional postural control during load carriage.

Although there is increasing interest in investigating how backpack loads affect children’s biomechanics, the existing literature has tended to investigate changes that follow a load through specific descriptions and paid little to no attention to intervention-based strategies. There are some studies looking at whether an explicit exercise program can attenuate the biomechanical effects of loading and increase functional postural resilience. Filling this gap will be crucial for the implementation of evidence-based interventions to alleviate mechanical overload and encourage healthier movement patterns in schooling (15, 16).

Thus, this study seeks to assess the influence of a 16-week structured exercise program on plantar pressure distribution and postural resilience of schoolchildren while walking with a schoolbag. In particular, the purpose of this study is to verify if the intervention ameliorates peak plantar pressure and the performance of the gait mechanics in a load-bearing condition and how well it may induce a more effective, stable manner.

## 2 Methods

### 2.1 Study design

This study employed a controlled, non-randomized experimental design with pre- and post-intervention assessments. Participants were recruited using a random sampling approach from primary schools located in five geographically distinct Albanian cities (Tirana, Elbasan, Shkodër, Vlorë, and Pogradec). Following recruitment, participants were assigned to either the experimental or control group according to the study implementation protocol. Because individual random allocation was not performed, the study should be considered a controlled non-randomized intervention study.

### 2.2 Participants

A total of 147 school-aged children participated in the study. Subjects were divided into an experimental group (*n* = 74) and a control group (*n* = 73). Participants were recruited from primary schools and fell into an age range characterized by ongoing neuromuscular and postural development. All participants were healthy and ambulatory enough to perform walking tasks independently. Patients had no diagnosed neurological and/or musculoskeletal disorders, no recent lower limb injuries, no structural gait abnormalities, and no participation in a supplementary training service other than regular school activities.

Baseline anthropometric characteristics, including age, body height, body weight, and body mass index (BMI), were comparable between the experimental and control groups (Table 1). Therefore, BMI was not considered a confounding factor in the primary between-group comparisons and was not included as a covariate in the statistical analyses.

**Table 1:** Baseline demographic and anthropometric characteristics of the study participants.

Variable	Control (*n* = 73)	Experimental (*n* = 74)	*p*-value
Age (years), mean ± SD	13.12 ± 1.79	12.58 ± 1.05	0.027
Male, *n* (%)	29 (39.7)	35 (47.3)	0.448
Female, *n* (%)	44 (60.3)	39 (52.7)	
Height (cm), mean ± SD	162.30 ± 10.02	161.22 ± 8.71	0.484
Body mass (kg), mean ± SD	58.27 ± 13.21	53.34 ± 11.26	0.016
Body mass index (kg/m^2^), mean ± SD	21.99 ± 4.08	20.46 ± 3.89	0.022

Data are presented as mean ± standard deviation (SD) unless otherwise indicated. Continuous variables were compared using independent-samples *t*-tests, whereas sex distribution was compared using the chi-square test. Statistical significance was set at *p* < 0.05.

### 2.3 Intervention program

Participants allocated to the experimental group completed a standardized 16-week exercise program designed to improve postural alignment, neuromuscular coordination, balance, lower-limb muscle strength, and gait stability during load carriage. The intervention was developed in accordance with the World Health Organization’s recommendations for children and adolescents and was delivered by certified physical education instructors under the supervision of researchers from the Sports University of Tirana. The intervention consisted of three supervised sessions per week (Monday, Wednesday, and Friday), totaling 48 sessions over the 16-week study period. Each session lasted approximately 60 min and followed a standardized structure comprising a 10–15-min warm-up, a 40–45-min main exercise phase, and a 5–10-min cool-down. Exercise intensity and complexity progressed systematically throughout the intervention according to predefined progression criteria while considering the participants’ age, biological sex, and baseline physical fitness.

Attendance was monitored throughout the intervention by the supervising instructors to ensure adherence to the standardized training schedule. All participants completed the intervention and were included in the final analysis.

**Figure 1:**
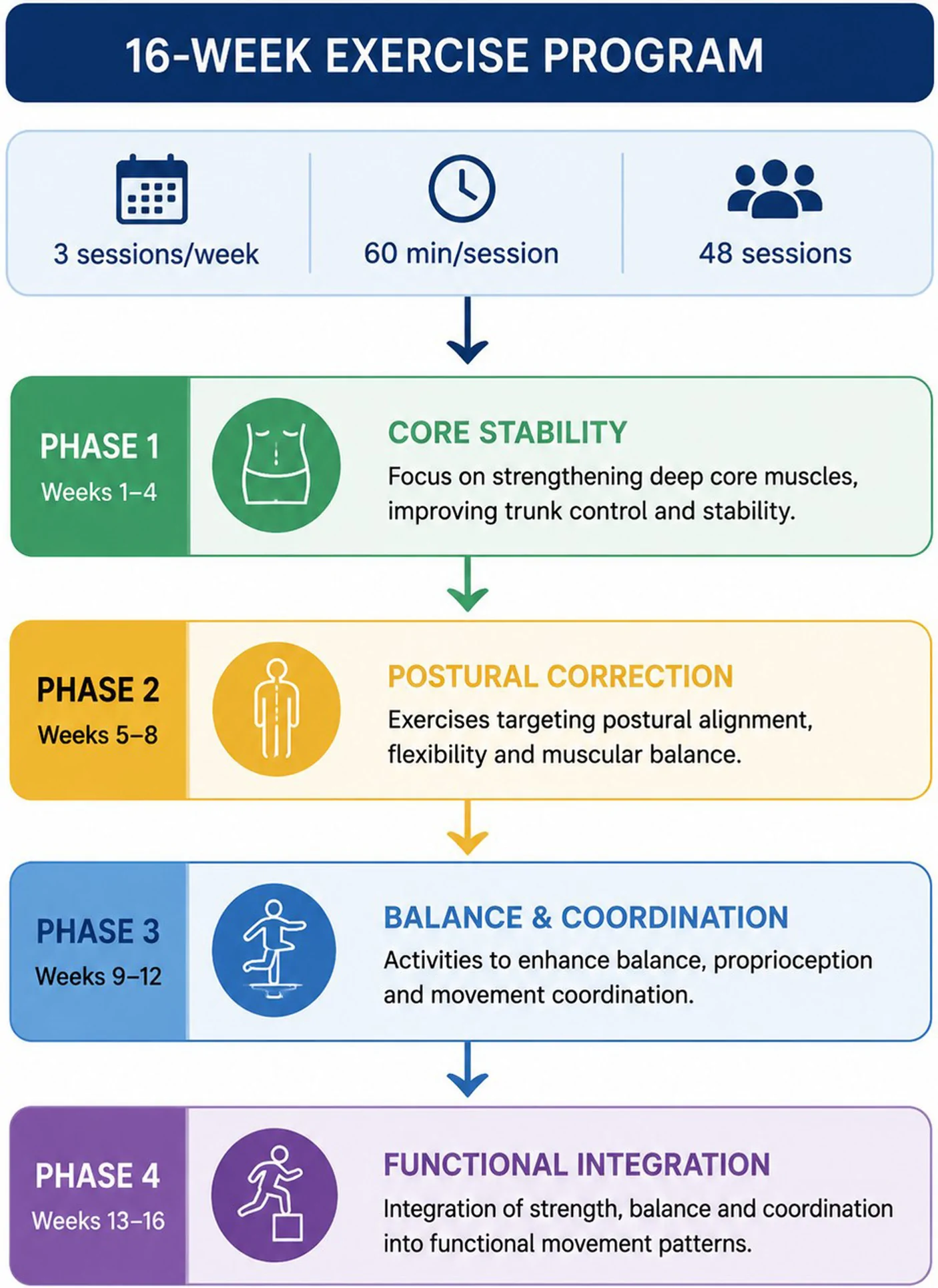
Overview of the 16-week structured exercise intervention implemented in the experimental group. The program consisted of three supervised 60-min sessions per week (48 sessions total) and progressed through four sequential phases targeting foundational core stability, postural correction and muscular rebalancing, balance and coordination, and functional integration with dynamic strength. Detailed exercise descriptions are provided in Supplementary Appendix 1 and Supplementary Table S2.

The program was organized into four progressive phases focusing on (1) foundational core stability, (2) postural correction and muscular rebalancing, (3) balance and neuromuscular coordination, and (4) functional integration and dynamic strength. A detailed description of the intervention protocol, including exercise selection, session structure, progression criteria, and physiological objectives, is provided in Supplementary Appendix 1 to facilitate study replication. A graphical overview of the intervention is presented in Figure 1, whereas a detailed description of the intervention protocol, including exercise selection, session structure, progression criteria, and physiological objectives, is provided in Supplementary Appendix 1 and Supplementary Table S2 to facilitate replication of the study. The protocol was developed based on the literature specific to this age group (17–22). Participants allocated to the control group attended only the regular school physical education curriculum implemented in Albanian public schools. Physical education lessons were delivered by certified physical education teachers during regular school hours and generally included warm-up activities, general fitness exercises, running drills, flexibility exercises, fundamental motor skill development, team games, and recreational sports. The control curriculum did not include any structured exercises specifically targeting postural alignment, core stabilization, balance training, neuromuscular coordination, or gait mechanics during schoolbag load carriage.

### 2.4 Instrumentation

Measurement of plantar pressure was performed with the T&T Medilogic^®^ System, a high-resolution instrument used to record force distribution on the surface of the plant. Quantitative measures of pressure magnitude (*N*/cm^2^) are also provided, and load maps during dynamic activities can be generated using the system. For biomechanical responses to functional conditioning in pediatric populations, this is a reliable and valid tool.

All plantar pressure assessments were performed by the same trained investigator using the T&T Medilogic^®^ system to ensure methodological consistency and reduce inter-operator variability. Because the measurements required specialized equipment and operator expertise, blinding of the outcome assessor to group allocation was not feasible. However, standardized measurement procedures were applied for all participants under identical testing conditions.

### 2.5 Testing procedures

All biomechanical assessments were performed under standardized testing conditions before and after the 16-week intervention in the Biomechanics Laboratory of the Sports University of Tirana. Participants completed walking trials at their self-selected comfortable speed while barefoot across the T&T Medilogic pressure measurement platform. Each walking trial was performed along an approximately 30-m walking pathway (out-and-back), allowing participants to establish a natural gait pattern before and after crossing the pressure platform. Each participant completed three valid walking trials, separated by standardized rest intervals of approximately 5–10 min to minimize the effects of fatigue.

During the walking-with-schoolbag assessment, participants carried their own double-strap school backpack containing the educational materials required for that school day. The schoolbag load corresponded to a minimum of 10% of each participant’s body mass and, in some cases, exceeded this value depending on the number and weight of the required textbooks and school materials. This approach was intentionally adopted to reproduce authentic school conditions rather than an artificially standardized laboratory loading protocol. Establishing a minimum load corresponding to 10% of body mass ensured consistency with commonly recommended schoolbag guidelines, while allowing natural day-to-day variation in school materials reflected the typical mechanical demands experienced by schoolchildren during routine school activities (13, 15). This approach was adopted to reflect typical school conditions while maintaining a minimum loading threshold consistent with current recommendations for school-aged children. All pre- and post-intervention assessments were conducted using the same testing protocol, equipment, environmental conditions, and measurement procedures to ensure consistency and comparability of the recorded data.

### 2.6 Outcome measures

The main outcome variable was maximum plantar pressure (*N*/cm^2^) during walking with load, which indicates localized mechanical stress and load distribution efficiency. This parameter measures the ability of the musculoskeletal system under external load during locomotion. Secondary outcomes were postural resilience indices based on the changes in plantar pressure patterns and reductions in peak loading values after the intervention.

### 2.7 Statistical analysis

Descriptive statistics were calculated for all study variables and are presented as means and standard deviations (SD). Data normality was assessed using the Kolmogorov–Smirnov and Shapiro–Wilk tests, while homogeneity of variances was evaluated using Levene’s test. Although both normality tests indicated statistically significant deviations from a normal distribution (*p* < 0.001), parametric analyses were considered appropriate because of the relatively large and balanced sample sizes in both groups (*n* = 73 and *n* = 74), the robustness of parametric procedures to moderate violations of normality, and the homogeneity of variances confirmed by Levene’s test (*p* > 0.05). Furthermore, inspection of skewness and kurtosis values provided additional support for evaluating the suitability of parametric statistical methods. Observed skewness values ranged from −2.19 to 2.08, whereas kurtosis values ranged from 2.23 to 6.17. Although two variables slightly exceeded the conventional skewness threshold of ±2, kurtosis values remained below the commonly accepted limit of 7. Considering the relatively large and balanced sample sizes, the homogeneity of variances confirmed by Levene’s test, and the robustness of parametric tests to moderate deviations from normality, parametric analyses were considered appropriate (23, 24). Within-group changes between pre- and post-intervention measurements were evaluated using paired-samples *t*-tests, whereas between-group comparisons were performed using independent-samples *t*-tests. The effects of the intervention over time were examined using repeated-measures analysis of variance (ANOVA), including the main effects of time and group, as well as the time × group interaction. A two-way ANOVA was additionally conducted to examine potential gender-related effects. Effect sizes were calculated using Cohen’s *d* to quantify the magnitude of observed differences. Statistical significance was established at *p* < 0.05 for all analyses. All statistical analyses were performed using IBM SPSS Statistics (Version XX, IBM Corp., Armonk, NY, United States).

### 2.8 Sample size and power analysis

A sample size estimation was performed using G*Power software (version 3.1) for a two-tailed comparison between two independent groups. The calculation was based on a medium standardized effect size (Cohen’s d = 0.50), an alpha level of 0.05, statistical power of 0.80, and an allocation ratio close to 1:1. The analysis indicated that a minimum of 128 participants was required. Therefore, the final sample of 147 participants (experimental group: *n* = 74; control group: *n* = 73) exceeded the minimum required sample size and was considered adequate for detecting a medium intervention effect. Based on the final sample size, the achieved statistical power was approximately 0.85.

## 3 Results

### 3.1 Baseline characteristics

Baseline demographic and anthropometric characteristics are summarized in Table 1. The experimental and control groups were comparable with respect to sex distribution and body height (*p* > 0.05). However, statistically significant differences were observed for age, body mass, and BMI (*p* < 0.05). These baseline differences were considered when interpreting the intervention findings.

All enrolled participants completed the 16-week study and were included in the final statistical analyses. No participant withdrew during the intervention period, and complete pre- and post-intervention data were available for all participants. Attendance at the exercise sessions was monitored continuously by the supervising physical education instructors using routine attendance records to ensure compliance with the intervention protocol. Throughout the study, no exercise-related adverse events, musculoskeletal injuries, or other safety concerns were observed or reported. These findings indicate that the intervention was well tolerated, feasible to implement within the school setting, and safe for school-aged children. Attendance at the exercise sessions was monitored continuously by the supervising physical education instructors throughout the intervention to ensure adherence to the exercise protocol.

### 3.2 Plantar pressure while on schoolbag during walking

Findings of maximal plantar pressure during schoolbag walking revealed robust group-specific adaptive changes across 16 weeks of the intervention. Plantar pressure decreases between pre- and post-treatment in the experimental condition (54.18 ± 11.84 to 52.40 ± 12.41 N/cm^2^) were statistically significant (*p* = 0.010; d = 0.31), as shown in the descriptive statistics (Table 2). The change was relatively small and insignificant over the control group (57.09 ± 9.42 to 56.40 ± 9.67 N/cm^2^; *p* = 0.37), and this suggests that the natural adaptation to load carriage is suppressed without focus-training.

**Table 2:** Descriptive statistics for maximum plantar pressure when walking with schoolbag (*N*/cm^2^).

Group	Pre (M ± SD)	Post (M ± SD)	Δ (Post–Pre)	Cohen’s *d*	*t*(df)	*p*-value
Control (*n* = 73)	57.09 ± 9.42	56.40 ± 9.67	−0.69	−0.11	−0.91 (72)	0.37
Experimental (*n* = 74)	54.18 ± 11.84	52.40 ± 12.41	−1.78	−0.31	−2.63 (73)	0.010*

*p* < 0.05; * = statistically significant.

When the experimental group and the control group showed differences in lower level of reduction, it indicates the effect of treatment; the scale changes, *Δ* = −1.78 N/cm^2^ vs. negative Δ = −0.69 N/cm^2^ for the control group. Difference was defined between-groups (*t*(145) = 2.45, *p* = 0.016). This sequence signifies improved mechanical ability and hence the intervention led to a more effective redistribution of ground reaction forces on the plantar surface.

**Figure 2:**
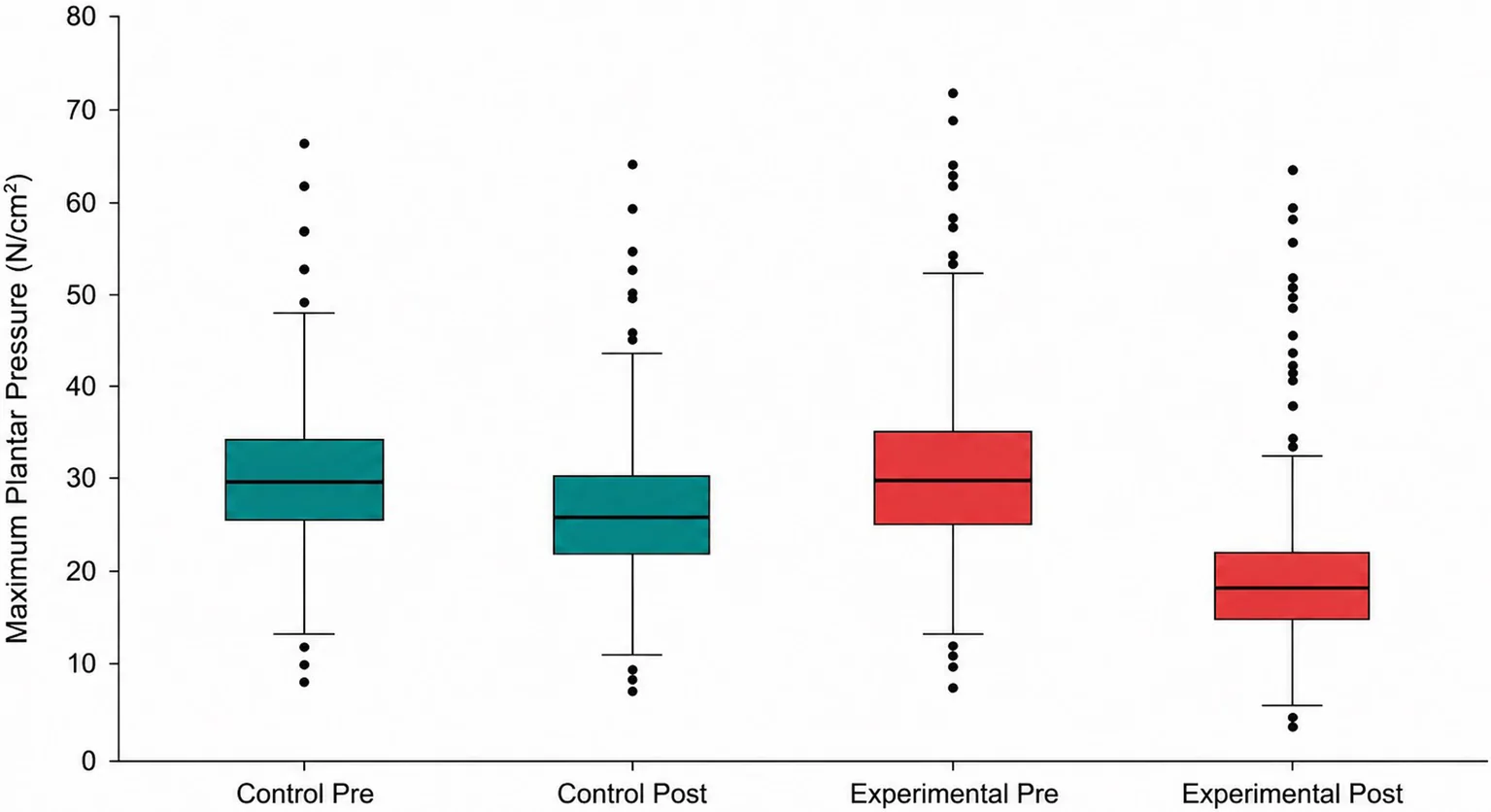
Maximum plantar pressure during walking with a schoolbag before and after the 16-week intervention in the control and experimental groups. Boxplots show the median (line inside the box), interquartile range (box), whiskers (1.5 × IQR), and outliers (●). Control group (*n* = 73); experimental group (*n* = 74).

Figure 2 presents the distribution of maximum plantar pressure (*N*/cm^2^) during normal walking with a schoolbag before and after the 16-week intervention in both study groups. The control group showed minimal changes between the pre- and post-intervention assessments, whereas the experimental group demonstrated a noticeable reduction in maximum plantar pressure following completion of the intervention program. The boxplots illustrate the distribution, central tendency, variability, and individual outliers for each assessment condition.

Comparison between the experimental and control groups demonstrated a more homogeneous plantar pressure distribution following the intervention in the experimental group. The most evident changes were observed in the heel and forefoot regions, where peak pressure values were reduced, indicating a more balanced transfer of body weight during walking. In contrast, the control group showed minimal changes in plantar pressure distribution between the pre- and post-intervention assessments, suggesting that the observed biomechanical adaptations were primarily associated with the structured exercise program.

This indicates that the experimental group showed a larger percentage decrease in plantar pressure (−3.3%) than the control group (−1.2%), suggesting that a better-structured approach leads to a greater reduction in plantar loading during walking with a schoolbag rather than merely reflecting small differences in numerical performance.

**Figure 3:**
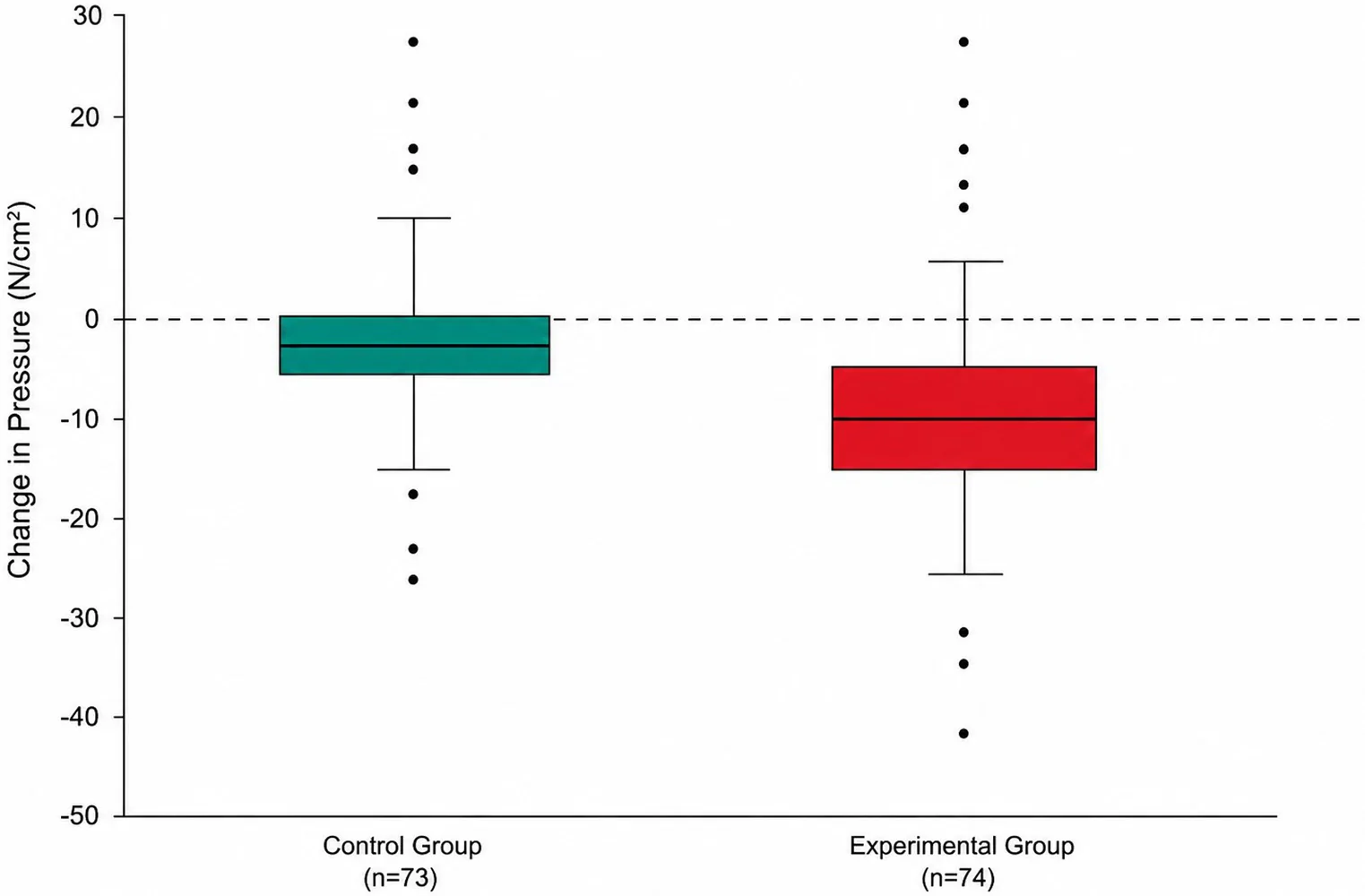
Change in maximum plantar pressure (post − pre). Negative values indicate a reduction in maximum plantar pressure after the intervention, whereas positive values indicate an increase. Boxplots display the median (horizontal line), interquartile range (box), whiskers (1.5 × IQR), and individual outliers (●). Control group (*n* = 73); Experimental group (*n* = 74).

Figure 3 presents the individual changes in maximum plantar pressure between the post- and pre-intervention assessments (Post − Pre) for both study groups. The control group exhibited only small changes centered around zero, indicating minimal variation in plantar pressure over the study period. In contrast, the experimental group demonstrated a marked shift toward negative values, reflecting a greater reduction in maximum plantar pressure following the 16-week exercise intervention. The distribution of change scores also indicates a more consistent improvement among participants in the experimental group compared with the control group.

### 3.3 Optimization of plantar pressure from a biomechanical perspective

The reduction of plantar pressure in the experimental group is associated with structural enhancement of load bearing gait owing to biomechanical organization. Lower peak values mean less local mechanical force (i.e., less loading of plantar surface) and a better force redistribution along the plantar surface. Accordingly, this plasticity can probably be said to correspond to better alignment/ neurology of the trunk as well as less overloading of the anterior part of the front of the body in a backpack transfer context. There was also greater stability in transition of weight in stance mode and a less acute peak pressure attributed to better center of mass control. These changes collectively suggest that the intervention reinforced better stabilization and efficiency of the gait strategy at load and improved trunk stabilization and limb performance consolidation.

### 3.4 Weight-bearing symmetry

The intervention significantly improved weight-carrying symmetry, with more balanced weight distribution between both arms (Table 3). In the experimental group, there was a strong trend towards symmetrical loading (46.4 ± 4.1% vs. 49.2 ± 3.6%), with an average of an improvement of +2.8% (*p* < 0.001; d = 0.48). In contrast, the control group had just a very small non-significant increase (from 46.9 ± 3.7% to 47.6 ± 3.9%; *p* = 0.25).

**Table 3:** Descriptive statistics for weight-bearing symmetry (%).

Group	Pre (M ± SD)	Post (M ± SD)	Mean *Δ* (Post–Pre)	*t*(df)	*p*-value	Cohen’s *d*-value	Interpretation
Control (*n* = 73)	46.9 ± 3.7	47.6 ± 3.9	+0.7	1.15 (72)	0.25	0.13	Small, non-significant shift toward symmetry.
Experimental (*n* = 74)	46.4 ± 4.1	49.2 ± 3.6	+2.8	4.12 (73)	<0.001**	0.48	Moderate, significant improvement in bilateral load distribution.

Δ = post-intervention minus pre-intervention. Data are presented as mean ± standard deviation (SD). Statistical significance was set at *p* < 0.05. ** = *p* < 0.001. Cohen’s d values were interpreted as small (≈0.20), medium (≈0.50), and large (≈0.80).

Between-group comparison of the change scores demonstrated a statistically significant difference (*t*(145) = 3.78, *p* < 0.001), indicating that the greater improvement in weight-bearing symmetry observed in the experimental group was attributable to the intervention rather than to natural variation over time. The observed improvement suggests a more balanced bilateral distribution of plantar loading following the intervention, reflecting enhanced postural control during standing.

**Figure 4:**
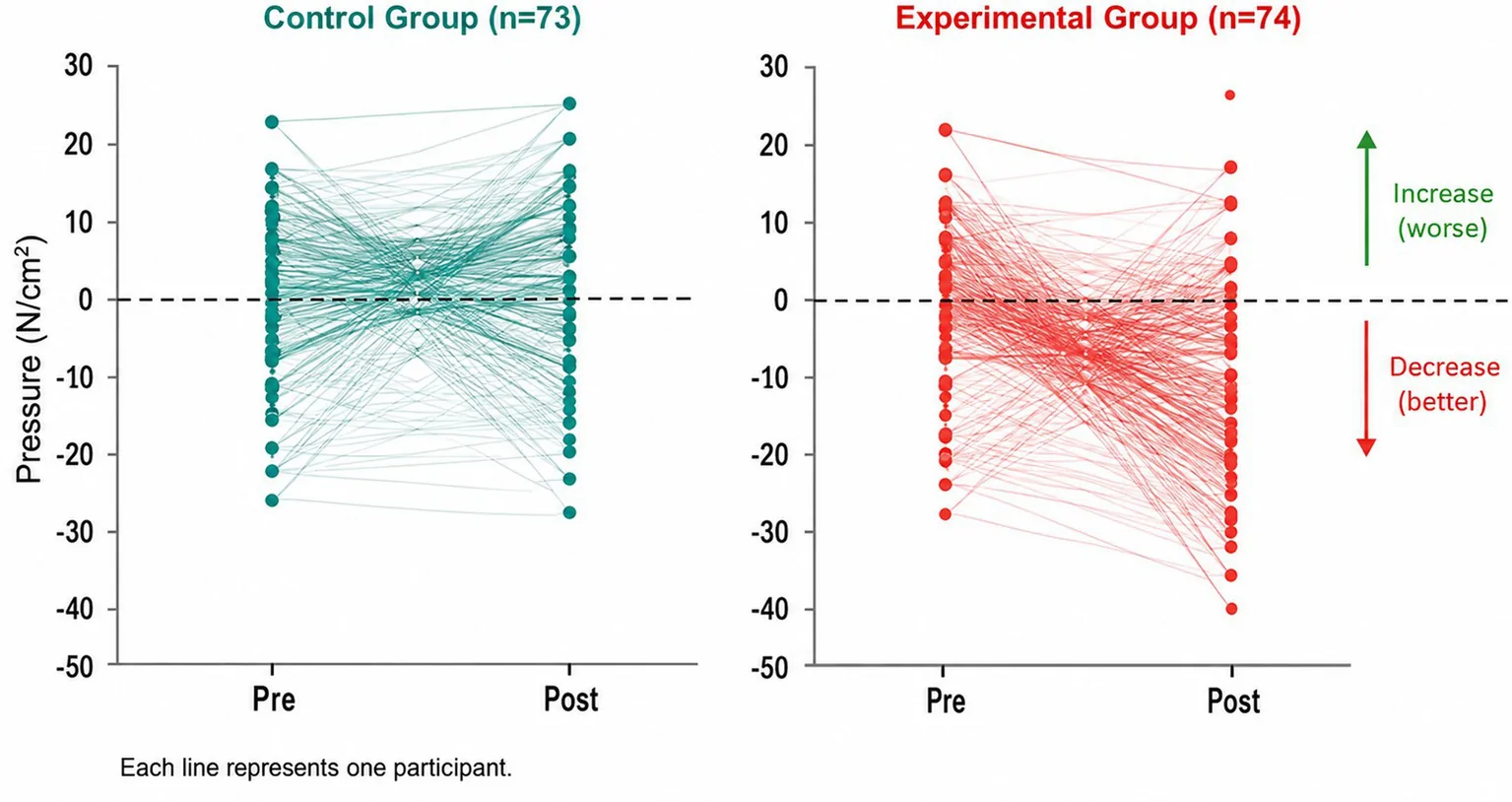
Individual changes in maximum plantar pressure.

Figure 4 presents the individual changes in maximum plantar pressure from pre- to post-intervention for participants in both the control and experimental groups. Each line represents one participant, connecting the pre- and post-intervention measurements. While the control group shows relatively small and mixed changes, the experimental group demonstrates a more pronounced downward trend, indicating a reduction in maximum plantar pressure following the intervention. Lower post-intervention values suggest an improvement in plantar pressure distribution, whereas increases indicate worsening.

The experimental group shows improved bilateral weight-bearing symmetry and function by receiving more positive change up to 50% bilateral load. These results show that the intervention affected mechanisms of neuromuscular control so that participants have a balanced distribution of vertical load. Improved symmetry suggests that this improves stabilization with greater of lumbopelvic motion stability and reduced compensatory load strategy.

### 3.5 ANOVA: the test for repeated measures—walking with a schoolbag

The normality test (Table 4), conducted before the inferential test, indicated significant deviations from normality for all variables (*p* < 0.001). But, due to the vast size of the sample and satisfactory values for skewness and kurtosis, the data were amenable for parametric analysis.

**Table 4:** Normality assessment of maximum plantar pressure during walking with a schoolbag before and after the intervention.

Normality tests							
Variable	Group	Kolmogorov-Smirnov^a^			Shapiro–Wilk		
		Statistic	df	Sig.	Statistic	df	Sig.
Walking with schoolbag – Pressure scale max N/cm^2^_Pre	control	0.209	73	0.000	0.743	73	0.000
	exp	0.225	74	0.000	0.762	74	0.000
Walking with schoolbag – Pressure scale max N/cm^2^_Post	control	0.182	73	0.000	0.755	73	0.000
	exp	0.209	74	0.000	0.815	74	0.000

a Lilliefors significance correction.

A statistically significant result (*p* < 0.05) indicates deviation from a normal distribution. Despite these findings, parametric analyses were retained because the study included relatively large and balanced groups, homogeneity of variances was confirmed by Levene’s test (*p* > 0.05), and parametric statistical methods are generally considered robust to moderate departures from normality assumption.

Repeated measures ANOVA (Table 5) revealed a significant main effect of intervention (*F* (1,145) = 49.881, *p* < 0.001), indicating a significant overall change in maximum plantar pressure between the pre- and post-intervention assessments. More importantly, a significant intervention × group interaction was observed (*F* (1,145) = 9.842, *p* = 0.002), demonstrating that the magnitude of change differed significantly between the experimental and control groups. These findings indicate that exercise intervention produced greater improvements than those attributable to natural changes over time alone.

**Table 5:** Repeated measures ANOVA for within-subject effects.

Source	Correction	Type III sum of squares	df	Mean square	*F*	*p*-value
Intervention	Sphericity Assumed	111.615	1	111.615	49.881	<0.001*
	Greenhouse–Geisser	111.615	1.000	111.615	49.881	<0.001*
	Huynh–Feldt	111.615	1.000	111.615	49.881	<0.001*
	Lower-bound	111.615	1.000	111.615	49.881	<0.001*
Intervention × Group	Sphericity Assumed	22.023	1	22.023	9.842	0.002*
	Greenhouse–Geisser	22.023	1.000	22.023	9.842	0.002*
	Huynh–Feldt	22.023	1.000	22.023	9.842	0.002*
	Lower-bound	22.023	1.000	22.023	9.842	0.002*
Error (intervention)	Sphericity Assumed	324.457	145	2.238	–	–
	Greenhouse–Geisser	324.457	145.000	2.238	–	–
	Huynh–Feldt	324.457	145.000	2.238	–	–
	Lower-bound	324.457	145.000	2.238	–	–

Repeated-measures analysis of variance (ANOVA) was performed to evaluate the effects of time (pre- vs. post-intervention), group (control vs. experimental), and their interaction on maximum plantar pressure when walking with a schoolbag. Greenhouse–Geisser, Huynh–Feldt, and Lower-bound corrections are presented for completeness. Because only two repeated measurements (pre- and post-intervention) were included, all correction methods produced identical results. Statistical significance was established at *p < 0.05.

The significant main effect of intervention indicates that maximum plantar pressure changed significantly over time across the study population. More importantly, the significant intervention × group interaction demonstrates that the experimental group experienced a significantly greater reduction in maximum plantar pressure than the control group, confirming the effectiveness of the 16-week exercise intervention.

To further examine between-group differences irrespective of the repeated measurements, a univariate analysis of variance (ANOVA) was performed (Table 6). The results demonstrated a statistically significant main effect of group, indicating that plantar pressure differed between the experimental and control groups following the intervention.

**Table 6:** Analysis of variance (ANOVA) for between-group differences in maximum plantar pressure.

Source	Type III sum of squares	df	Mean square	*F*	*p*-value
Intercept	889788.269	1	889788.269	3764.740	<0.001*
Group	878.868	1	878.868	3.709	0.050*
Error	34270.445	145	236.348	–	–

A univariate analysis of variance (ANOVA) was conducted to examine between-group differences in maximum plantar pressure. Statistical significance was established at *p < 0.05.

The ANOVA demonstrated a borderline statistically significant main effect of group (*F* (1,145) = 3.709, *p* = 0.050), indicating modest overall differences in maximum plantar pressure between the experimental and control groups. This finding should be interpreted alongside the significant intervention × group interaction observed in the repeated-measures ANOVA, which provides stronger evidence that the intervention influenced the magnitude of change over time. Additionally, a supplementary two-way ANOVA revealed no significant main effect of sex (*F* (1,143) = 0.185, *p* = 0.668) and no significant group × sex interaction (*F* (1,143) = 0.099, *p* = 0.753). These findings indicate that the effects of the intervention were comparable between male and female participants, suggesting that sex did not significantly influence the observed changes in maximum plantar pressure.

**Figure 5:**
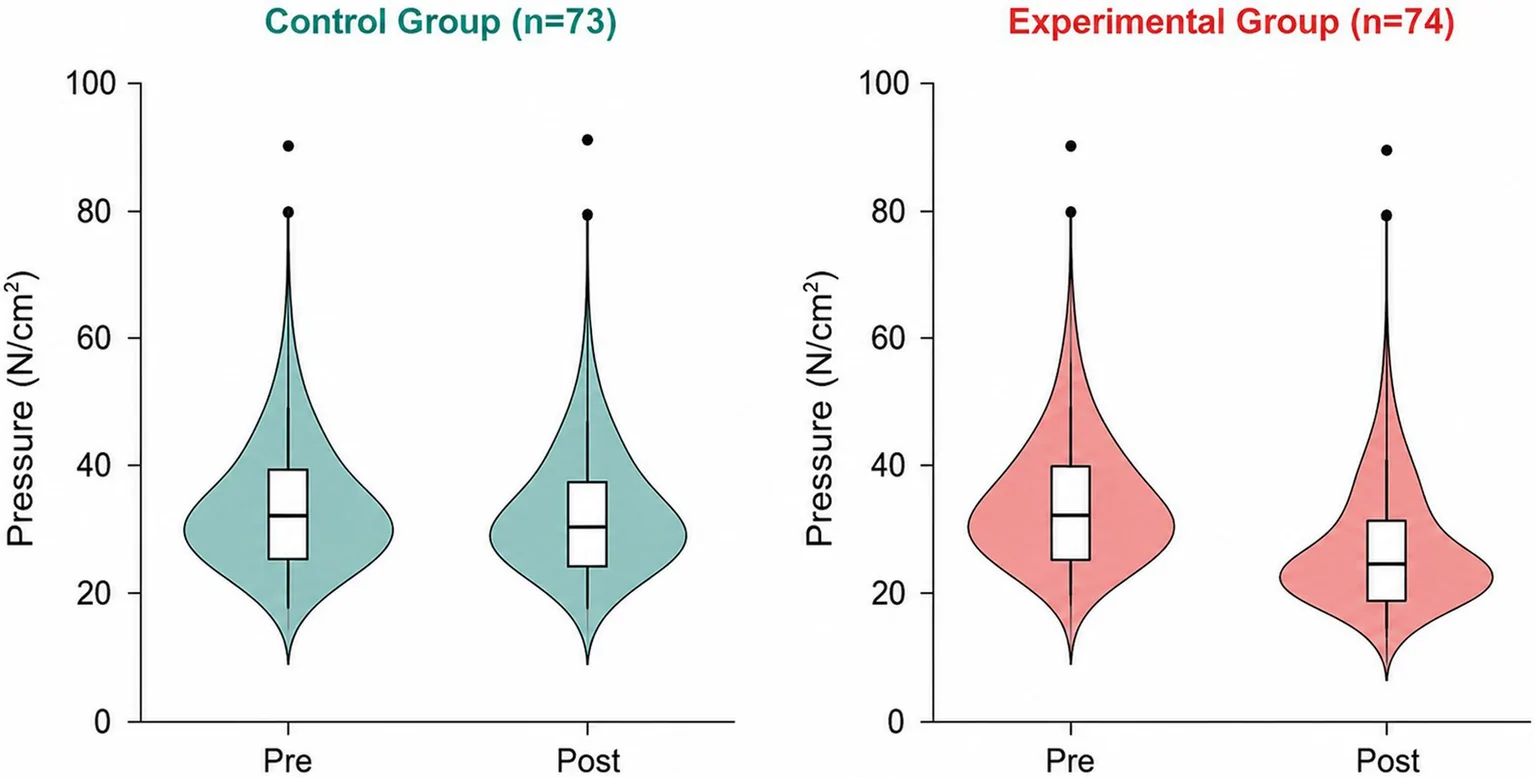
Distribution and density of maximum plantar pressure (pre vs. post). Violin plots illustrate the distribution and probability density of maximum plantar pressure values (*N*/cm^2^). The embedded boxplots display the median (horizontal line), interquartile range (box), and whiskers, while the violin shape represents the distribution of individual observations. Control group (*n* = 73); experimental group (*n* = 74).

Figure 5 presents the distribution and density of maximum plantar pressure during normal walking with a schoolbag before and after the intervention. In the control group, the overall distribution remained largely unchanged between the pre- and post-intervention assessments, indicating minimal variation in plantar pressure. In contrast, the experimental group demonstrated a clear shift of the distribution toward lower pressure values following the intervention, accompanied by a reduction in data dispersion. These findings complement the boxplot analyses and provide additional evidence of the intervention’s effectiveness in reducing plantar loading when walking with a schoolbag.

To complement the graphical presentation of plantar pressure distributions, descriptive and inferential statistics for maximum plantar pressure under the three testing conditions are summarized in Table 7. These analyses provide a quantitative comparison of pre- and post-intervention changes in both the control and experimental groups.

**Table 7:** Maximum plantar pressure under the three testing conditions before and after the 16-week intervention.

Condition	Group	Pre (Mean ± SD)	Post (Mean ± SD)	Δ (Post–Pre)	*p*-value	Cohen’s *d*
Standing	Control (*n* = 73)	12.71 ± 6.25	12.11 ± 6.42	−0.60	<0.001	−0.41
Standing	Experimental (*n* = 74)	13.50 ± 6.33	11.98 ± 6.90	−1.53	<0.001	−0.61
Walking	Control (*n* = 73)	51.67 ± 12.47	51.11 ± 12.79	−0.56	<0.001	−0.41
Walking	Experimental (*n* = 74)	52.41 ± 13.50	50.83 ± 14.11	−1.58	<0.001	−0.52
Walking with schoolbag	Control (*n* = 73)	57.09 ± 9.42	56.40 ± 9.66	−0.68	<0.001	−0.47
Walking with schoolbag	Experimental (*n* = 74)	54.18 ± 11.84	52.40 ± 12.41	−1.78	<0.001	−0.68

Data are presented as mean ± standard deviation (SD). Δ = post-intervention minus pre-intervention. Within-group comparisons were performed using paired-samples *t*-tests. Cohen’s *d* was calculated to estimate the magnitude of the intervention effect and interpreted as small (≈0.20), medium (≈0.50), and large (≈0.80). Statistical significance was established at *p* < 0.05.

**Figure 6:**
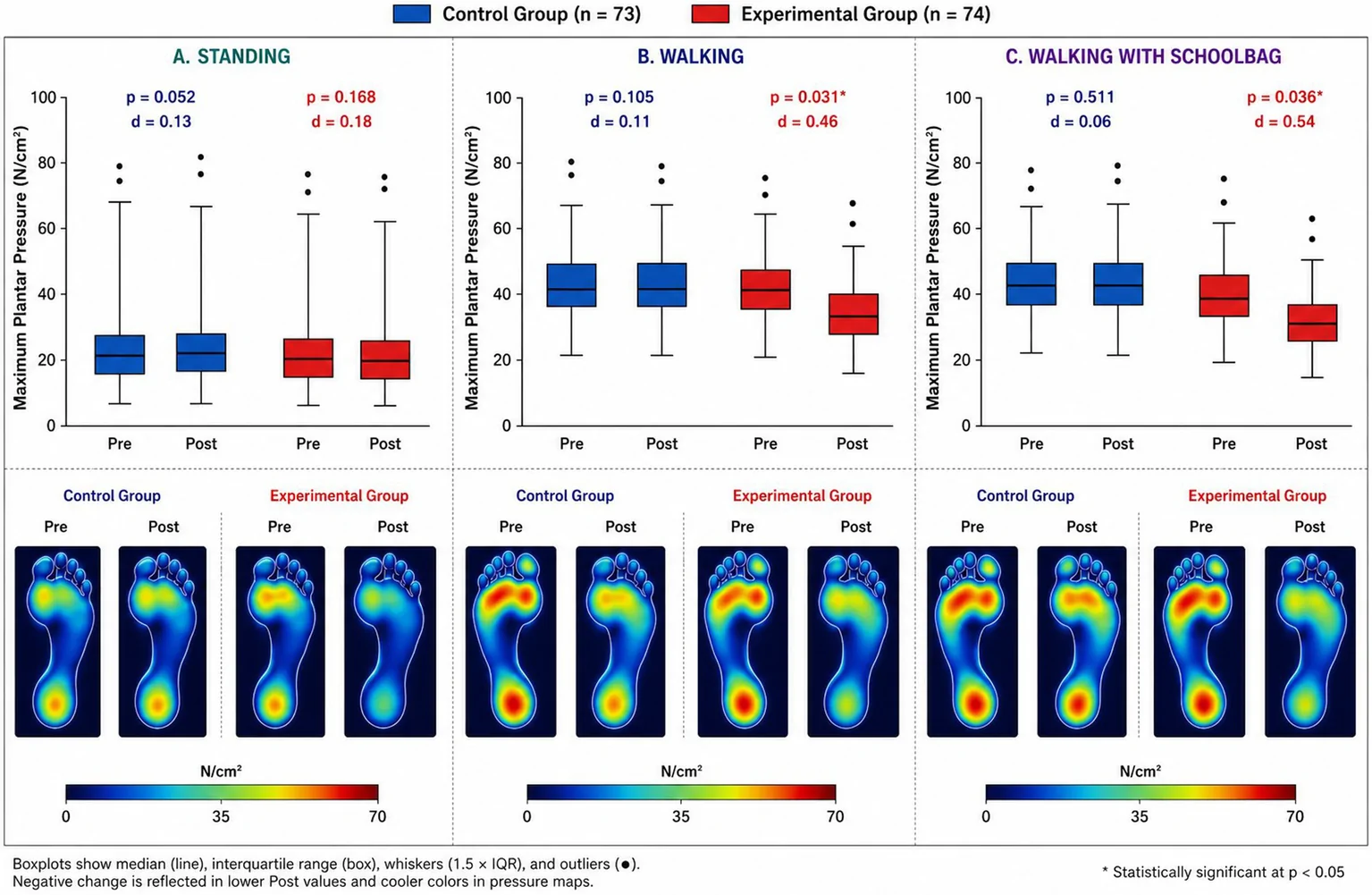
Summary of intervention effects on maximum plantar pressure maps during standing, walking, and walking with a schoolbag. The upper panels present boxplots of maximum plantar pressure measured under the three testing conditions (standing, walking, and walking with a schoolbag) in the control and experimental groups. Boxplots display the median (horizontal line), interquartile range (box), whiskers (1.5 × IQR), and individual outliers (●). The lower panels illustrate representative plantar pressure distribution maps obtained before and after the intervention. Warmer colors indicate higher plantar pressure, whereas cooler colors indicate lower plantar pressure. All boxplots were generated from the individual participant data included in the study.

As shown in Table 7, only minor changes were observed in the control group across the three testing conditions. In contrast, the experimental group demonstrated greater reductions in maximum plantar pressure following the 16-week intervention, with the largest improvement observed when walking with a schoolbag. These quantitative findings are consistent with the graphical distributions presented in Figures 5, 6.

Figure 6 presents a comprehensive overview of the intervention effects on plantar loading across the three experimental conditions. The upper row summarizes the distribution of maximum plantar pressure values obtained from all individual participants, while the lower row provides representative plantar pressure maps illustrating changes in pressure distribution before and after the intervention. Minimal changes were observed in the control group across all testing conditions. In contrast, the experimental group demonstrated a progressive reduction in maximum plantar pressure, particularly when walking with a schoolbag, accompanied by a more homogeneous plantar pressure distribution. Together, these findings provide complementary quantitative and visual evidence supporting the effectiveness of the 16-week exercise program in improving plantar loading patterns under increasing biomechanical demands.

The findings demonstrated that the 16-week structured exercise program resulted in significant improvements in plantar loading characteristics compared with the control condition. Participants in the experimental group exhibited greater reductions in maximum plantar pressure, accompanied by improved bilateral weight-bearing symmetry across the post-intervention assessments. These improvements were consistently observed across the standing, walking, and walking-with-schoolbag conditions and were further supported by the representative plantar pressure maps shown in Figure 6. In contrast, the control group demonstrated only minor changes over the study period, suggesting that participation in the regular school physical education curriculum alone was insufficient to induce comparable biomechanical adaptations.

Overall, these findings provide quantitative and visual evidence supporting the effectiveness of the structured exercise program in reducing plantar loading and improving weight-bearing symmetry during functional tasks performed under different loading conditions.

## 4 Discussion

The principal findings of the present study demonstrate that the 16-week structured exercise program significantly reduced maximum plantar pressure during walking with a schoolbag and improved weight-bearing symmetry compared with the control group. In contrast, only minimal changes were observed in the control group, suggesting that routine school activities alone were insufficient to induce comparable biomechanical adaptations. It is important to point out the minimal to non-significant changes in the control group, indicating a relatively weak adaptation to habitual load exposure lacking specific training. These findings strengthen prior findings suggesting that passive exposure to load carriage does not induce adequate neuromuscular adaptation in children (13, 25). The decrease in plantar pressure detected in the group reflects increased dispersal of ground reaction forces on the plantar surface. These findings suggest a more homogeneous redistribution of plantar loads when walking under external loading conditions. Load carriage is known to shift the center of mass anteriorly, resulting in compensatory trunk flexion, as well as increased loading in the forefoot and heel regions (26, 27). Nevertheless, the low peak pressure observed in the current study indicates that participants acquired adaptive strategies that reduced these changes. These results are consistent with previous research showing that structured exercise enhances postural alignment and decreases mechanical stress produced by a given load (12, 14).

A major finding is improved postural resilience, which is the capacity to maintain steady and effective movements over a higher mechanical load. The experimental group better attenuated pressure changes induced by load; thus, better center of mass control and stability of gait mechanics are suggested. By showing improved coordination between core musculature and lower-limb function because of the intervention, these conditions may result in better handling of external load. Additionally, enhancements in weight-bearing symmetry revealed in the results indicate improved symmetrical load patterns and bilateral coordination, suggesting functional compensatory strategies are likely in place and risk factors for injury are minimized (28). Research has reported similar adaptations, noting that proprioceptive and balance training can improve load handling capacity and ameliorate gait disturbances in children (8, 9). The results also suggest that muscular endurance and neuromuscular coordination can limit the biomechanical impact of schoolbag carriage and are important as they reduce muscle fatigue. Smaller plantar pressure values indicate less involvement of local compensatory forces and better uniformity of forces in the kinetic chain. Appropriate load distribution is linked to correct medial-lateral pressure transitions and reduced stress on joints, particularly at the ankle, knee, and lumbar spine, resulting in the stress load being mitigated on each site (29, 30). Moreover, enhanced bilateral symmetry is suggestive of improved lumbopelvic stability and optimal activation of stabilizing musculature leading to an improved dynamic balance and gait efficiency. These results are congruent with a biomechanical model that has shown that better neuromuscular control enables shock uptake and minimizes cumulative mechanical strain (31).

Importantly, this study adds to the emerging evidence that high backpack load is significantly associated with altered gait mechanics and risk for musculoskeletal disorders among children. Previous studies consistently reported the weight of schoolbags as an influential factor to spinal alignment, gait symmetry, and plantar pressure pattern (14–16). Other recent investigations have indicated that this overload of bearing loads may result in fatigue, decreased posture stability, and increased risk of injury, especially in the setting of poor physical conditioning (32, 33). The current study contributes to this literature by showing that targeted exercise intervention can mitigate these detrimental effects to support increased efficiency and stability of movement under load. Unlike studies with more limited descriptive analysis of load carriage, the present investigation of modifiable biomechanical responses provided by a structured workout suggests a modification effect due to training. These positive outcomes indicated that such neuromuscular adaptations were evident in almost immediate interventions and were observed to lead to functional gains in natural daily life.

The ability of participants to maintain lower plantar pressure levels under load indicates better adaptation to greater mechanical load rather than a decrease or improvement in motor performance. In a current systematic review (34, 35), it was observed that exercise-based interventions led to profound improvements in proprioception, balance, and functional load management in pediatric populations. A more applied view of these outcomes would mean that, from a school-based perspective, they are of notable relevance to the areas of public health and injury prevention. Children are frequently exposed to load carriage through schoolbags, creating a high demand for improved load handling, which may be key in reducing the risk of postural deviations and discomfort associated with chronic musculoskeletal disorders. The inclusion of well-planned exercise programs into school curricula can help to demonstrate that this approach can be applied in an effective and scalable manner to increase functional resilience and improve healthy musculoskeletal development (20, 36, 37). The absence of sex differences in the current study implies that these interventions may be effective in both male and female populations.

At a more global level, the improvement in the balance of plantar pressure and weight-bearing symmetry was a function of the intervention’s contribution to the increase in the mechanical efficiency of the whole gait pattern. These alterations represent a better synthesis of sensory feedback, neuromuscular guidance, and biomechanical coherence that allow children to adapt to external strain and remain stable and functional. These modifications are critical for normal motor development and to prevent the emergence of maladaptive movement patterns during growth (2, 3). The study concludes that children using load bearing benefit from a structured 16-week exercise training program to improve plantar pressure distribution and postural resistance. The intervention improved the ability to effectively transfer external load, decreased local mechanical stress, improved bilateral loading distribution, and created better balance in the gait process. These results highlight targeted exercise as a preventive and intervention for biomechanical problems relating to transportation in terms of carrying schoolbags in children.

Although previous studies have suggested that body composition and BMI may influence plantar loading patterns and postural control, baseline BMI did not differ significantly between the experimental and control groups in the present study (Table 1). Consequently, the observed improvements are more likely attributable to the structured exercise intervention than to differences in anthropometric characteristics. Future studies including broader BMI ranges and multivariable analytical approaches may further clarify the contribution of body composition to biomechanical adaptations during schoolbag carriage.

The schoolbag load was standardized to a minimum of 10% of each participant’s body mass but reflected the natural variation in educational materials carried on the testing day. Although previous studies have demonstrated that increasing backpack loads may produce progressively greater biomechanical alterations, the present approach was selected to reproduce typical school conditions rather than impose an artificial laboratory load. Consequently, some variability in mechanical demand may have existed among participants; however, all assessments were performed under identical pre- and post-intervention conditions for each participant, thereby minimizing its influence on the within-subject comparisons. Future studies employing multiple standardized loading conditions (e.g., 10, 15, and 20% of body mass) would further clarify dose–response relationships between external load and plantar pressure adaptations.

### 4.1 Study limitations

One limitation of the present study is the absence of individual random allocation to the intervention and control groups, which may have introduced a degree of selection bias. In addition, blinding of the outcome assessor was not feasible because all plantar pressure measurements were performed by the same trained investigator using specialized equipment. Although standardized measurement procedures were consistently applied to all participants to minimize measurement variability and ensure measurement consistency, the possibility of detection bias cannot be completely excluded. Baseline demographic and anthropometric comparisons are presented in Table 1 and were considered during the interpretation of the study findings. Future studies employing randomized allocation procedures and blinded outcome assessment, where feasible, are warranted to further strengthen methodological rigor and causal inference.

Despite these methodological limitations, the study provides novel evidence regarding the effects of a structured exercise program on plantar pressure distribution and load-related gait biomechanics in schoolchildren under standardized backpack-loading conditions.

## 5 Conclusion

The findings of the present study demonstrate that a 16-week structured exercise program significantly reduced maximum plantar pressure and improved weight-bearing symmetry during walking with a schoolbag in school-aged children. Compared with the control group, participants who completed the intervention exhibited more favorable plantar load distribution under functional loading conditions, suggesting improved adaptation to daily schoolbag carriage. These findings support the implementation of structured school-based exercise programs as a practical strategy to promote healthier load carriage biomechanics and reduce localized plantar loading during walking. Such interventions may contribute to the prevention of musculoskeletal overload associated with habitual schoolbag use and support healthy movement development during childhood.

### 5.1 Study impact

The present findings provide evidence that structured school-based exercise programs can positively influence plantar pressure distribution and weight-bearing symmetry during load carriage in children. These results support the integration of targeted neuromuscular and postural exercise programs into school physical education as a feasible strategy for promoting musculoskeletal health and reducing biomechanical stress associated with daily backpack use.

### 5.2 Future research

Future research should include randomized controlled trials with longer follow-up periods to determine whether the observed biomechanical improvements are maintained over time. Studies incorporating three-dimensional gait analysis, electromyography, and kinetic measurements would provide a more comprehensive understanding of the neuromuscular mechanisms underlying plantar pressure adaptations. In addition, future investigations should examine the influence of body composition, backpack load magnitude, and different age groups to further optimize school-based exercise interventions for children.

## Data Availability

The original contributions presented in the study are included in the article/Supplementary material, further inquiries can be directed to the corresponding author.
