# Supplementary material for: Load-carrying biomechanics in children: programed exercise enhances postural resilience and reduces plantar pressure under backpack load
Source: Front Public Health. 2026 Aug 11;14:1887693. doi: 10.3389/fpubh.2026.1887693 (PMC13503580; doi:10.3389/fpubh.2026.1887693)
Supplement: Supplementary file 1 [file Supplementary_file_1.pdf]

Supplementary Appendix 1

Detailed 16-Week Structured Exercise Intervention Protocol

Overview: The experimental group participated in a 16-week structured exercise intervention specifically designed to improve postural alignment, balance, neuromuscular coordination, lower-limb muscle strength, and gait stability during load carriage. The intervention was developed in accordance with the World Health Organization (WHO) recommendations for physical activity in children and adolescents and emphasized progressive motor skill development through age-appropriate exercises. The program was delivered by certified physical education instructors under the supervision of the research team from the Sports University of Tirana. All sessions were conducted in school sports facilities under standardized environmental conditions.

Implementation and Monitoring

The intervention consisted of 48 supervised exercise sessions delivered over 16 consecutive weeks (three sessions per week: Monday, Wednesday, and Friday). Each session lasted 60 minutes.

Attendance was recorded at every session using standardized attendance logs. Exercise technique, participant engagement, and safety were continuously monitored by the instructors throughout the intervention. Participants demonstrating fatigue, discomfort, or signs of excessive physical strain were instructed to discontinue exercise until full recovery. No serious adverse events or musculoskeletal injuries were recorded during the intervention period.

Session Structure

Each exercise session followed an identical standardized format.

Session Component	Duration	Description
Warm-up	10–15 min	Light jogging, rhythmic mobility exercises, and dynamic stretching (100–120 bpm).
Main exercise phase	40–45 min	Progressive exercises targeting postural control, core stability, balance, coordination, lower-limb strength, and functional movement patterns.
Cool-down	5–10 min	Static and dynamic stretching emphasizing spinal, hip, and shoulder flexibility.

Exercise intensity increased progressively throughout the intervention according to predefined objectives while respecting each participant's age, biological sex, and baseline physical fitness.

External resistance, when used (e.g., elastic bands or light external loads), never exceeded 10% of the participant's body mass, in accordance with pediatric exercise safety recommendations.

Phase 1 (Weeks 1–4): Foundational Core Development

Objective: To establish trunk stability and deep core muscle activation necessary for maintaining neutral spinal alignment and static postural control.

Main exercises

- Front plank
- Glute bridge
- Superman exercise
- Alternating arm-leg extensions
- Dynamic movement activation through low-intensity play activities
- Hip flexor flexibility exercises
- Lumbar mobility exercises

Primary physiological targets

- Core stability
- Lumbopelvic control
- Deep trunk muscle activation
- Static balance

Expected outcomes

- Improved trunk control
- Enhanced spinal stability
- Reduced postural asymmetry

Phase 2 (Weeks 5–8): Postural Correction and Muscular Rebalancing

Objective: To improve shoulder positioning and strengthen the posterior trunk musculature while reducing excessive thoracic kyphotic posture.

Main exercises

- Scapular retraction drills
- Wall-based postural correction exercises
- Isometric wall holds
- Elastic-band shoulder stabilization exercises
- Functional core stabilization exercises

Primary physiological targets

- Scapulothoracic stability
- Postural muscle endurance
- Trunk muscle balance
- Proprioception

Expected outcomes

- Improved upright posture
- Better head–shoulder alignment
- Improved postural awareness

Phase 3 (Weeks 9–12): Balance and Neuromuscular Coordination

Objective: To improve proprioceptive control, dynamic balance, and movement coordination during functional activities.

Main exercises

- Single-leg stance tasks
- Straight-line walking
- Proprioceptive exercises using visual and tactile feedback
- Controlled single-leg hopping
- Relay-based balance games
- Upper- and lower-limb coordination drills

Primary physiological targets

- Dynamic balance

- Neuromuscular coordination
- Lateral trunk stability
- Proprioception

Expected outcomes

- Improved balance control
- Enhanced gait stability
- Reduced compensatory movement patterns
- Improved movement accuracy

Phase 4 (Weeks 13–16): Functional Integration and Dynamic Strength

Objective: To integrate postural stability, muscular strength, and coordination into complex functional movement patterns representative of daily activities.

Main exercises

- Controlled squats
- Step-up exercises
- Walking with light loads ($\leq$10% body mass)
- Elastic-band shoulder stabilization
- Dynamic plank variations
- Mountain climbers
- Controlled jumping drills
- Functional running games with multidirectional movement

Primary physiological targets

- Functional strength
- Dynamic stability
- Movement coordination
- Functional endurance

Expected outcomes

- Improved functional gait performance
- Enhanced dynamic postural stability
- Improved movement efficiency

Supplementary Table S1.

Summary of the 16-Week Structured Exercise Intervention Protocol

Phase	Weeks	Primary Objective	Main Physiological Components	Expected Outcomes
Phase 1	1–4	Foundational core development	Core stability, trunk control, flexibility	Improved spinal stability and postural control
Phase 2	5–8	Postural correction and muscular rebalancing	Strength, proprioception, postural endurance	Improved upright posture and muscular symmetry
Phase 3	9–12	Balance and neuromuscular coordination	Balance, coordination, dynamic stability	Improved gait control and reduced compensatory movements
Phase 4	13–16	Functional integration and dynamic strength	Strength, coordination, functional endurance	Improved gait efficiency and functional movement performance

Quality Assurance

To ensure intervention fidelity, all sessions were supervised by the same team of certified physical education instructors using standardized instructional procedures. Exercise progression followed predefined criteria throughout the intervention, and participant attendance, exercise execution, and safety were documented after every session.
