# Supplementary material for: Load-carrying biomechanics in children: programed exercise enhances postural resilience and reduces plantar pressure under backpack load
Source: Front Public Health. 2026 Aug 11;14:1887693. doi: 10.3389/fpubh.2026.1887693 (PMC13503580; doi:10.3389/fpubh.2026.1887693)
Supplement: Supplementary file 2 [file Supplementary_file_2.pdf]

Supplementary Table S2

Operational Parameters of the 16-Week Exercise Intervention Protocol

Phase	Exercise Category	Representative Exercises	Weekly Frequency	Session Duration	Progression Strategy	External Load
**Phase 1 (Weeks 1–4)**	Core stabilization	Front plank, glute bridge, superman, alternating arm–leg extensions	3 sessions /week	60 min	Progressive increase in exercise complexity and neuromuscular control	Body weight only
**Phase 2 (Weeks 5–8)**	Postural correction and muscular rebalancing	Scapular retraction, wall-based isometric exercises, elastic-band shoulder stabilization, functional core exercises	3 sessions /week	60 min	Progressive increase in postural control demands and muscular endurance	Elastic bands (light resistance)
**Phase 3 (Weeks 9–12)**	Balance and neuromuscular coordination	Single-leg stance, straight-line walking, proprioceptive exercises, relay balance games, controlled hopping	3 sessions /week	60 min	Progressive reduction of external support and increased task complexity	Body weight only
**Phase 4 (Weeks 13–16)**	Functional integration and dynamic strength	Squats, step-ups, walking with schoolbag/light load, dynamic plank variations, mountain climbers, multidirectional running games	3 sessions /week	60 min	Progressive integration of strength, balance, coordination, and functional movement patterns	

Note: Each exercise session consisted of a standardized warm-up (10–15 min), a main exercise phase (40–45 min), and a cool-down (5–10 min). Exercise progression was individualized

according to the participant's age, biological sex, motor performance, and technical execution while maintaining the same training objectives across the intervention period. Whenever external resistance was incorporated, the applied load did not exceed 10% of the participant's body mass, in accordance with pediatric exercise safety recommendations.

Phase 1 (Weeks 1–4): Foundational Core Development

Objective: To establish trunk stability, activate the deep core musculature, improve postural alignment, and develop the neuromuscular foundation required for subsequent phases of the intervention.

Exercise Prescription

Exercise	Sets	Repetitions	Rest	Progression	Primary Target
Front plank	3	20–30 s	30 s	Increase holding time	Core stability
Side plank	3	20–30 s/side	30 s	Increase holding time	Lateral trunk stability
Glute bridge	3	12	30 s	Improve movement control	Gluteal activation
Superman	3	12	30 s	Increase movement accuracy	Back extensor strength
Bird-dog	3	10/side	30 s	Improve trunk stability	Neuromuscular control
Dead bug	3	10/side	30 s	Increase coordination	Lumbopelvic control
Alternating arm–leg extensions	3	10/side	30 s	Improve movement quality	Core coordination
Hip flexor stretching	2	20 s/side	15 s	Increase flexibility	Hip mobility
Lumbar mobility exercises	2	10	20 s	Increase range of motion	Spinal mobility

Phase Progression: Exercise difficulty was progressively increased by improving exercise technique, increasing isometric holding time, enhancing neuromuscular control, and optimizing movement quality while maintaining age-appropriate exercise intensity.

Phase 2 (Weeks 5–8): Postural Correction and Muscular Rebalancing

Objective: To improve postural alignment, strengthen the postural musculature, enhance scapular stability, and restore muscular balance while maintaining trunk stability developed during Phase 1.

Exercise Prescription

Exercise	Sets	Repetitions	Rest	Progression	Primary Target
Scapular retraction drills	3	15	30 s	Increase movement quality	Scapular stability
Wall angels	3	12	30 s	Increase range of motion	Postural alignment
Wall-based postural correction	3	12	30 s	Improve posture control	Postural awareness
Isometric wall hold	3	20–30 s	30 s	Increase holding time	Postural endurance
Elastic-band shoulder stabilization	3	15	45 s	Increase band tension gradually	Shoulder stability
Elastic-band rows	3	15	45 s	Increase movement control	Upper-back strength
Functional core stabilization	3	12	30 s	Increase exercise complexity	Core stability
Thoracic mobility exercises	2	10	20 s	Increase mobility	Thoracic mobility

Phase Progression: Exercise difficulty was progressively increased by improving postural control, muscular endurance, exercise precision, and elastic-band resistance while maintaining correct technique.

Phase 3 (Weeks 9–12): Balance and Neuromuscular Coordination

Objective: To improve dynamic balance, proprioception, neuromuscular coordination, and gait stability through progressively more challenging balance and movement-control exercises.

Exercise Prescription

Exercise	Sets	Repetitions	Rest	Progression	Primary Target
Single-leg stance	3	20–30 s/leg	30 s	Reduce external support	Static balance
Tandem stance	3	20–30 s	30 s	Increase holding time	Postural control
Straight-line walking	3	10 m	30 s	Increase precision	Gait control
Heel-to-toe walking	3	10 m	30 s	Reduce visual cues	Dynamic balance
Proprioceptive balance exercises	3	30 s	30 s	Increase task complexity	Proprioception
Controlled single-leg hopping	3	8/leg	45 s	Increase movement accuracy	Dynamic stability
Relay balance games	3	45–60 s	45 s	Increase coordination demands	Agility & coordination
Upper- and lower-limb coordination drills	3	30 s	30 s	Increase movement speed	Neuromuscular coordination

Phase Progression: Exercise difficulty was progressively increased by reducing external support, increasing movement complexity, improving coordination demands, and enhancing dynamic balance while maintaining proper movement quality and safety.

Phase 4 (Weeks 13–16): Functional Integration and Dynamic Strength

Objective: To integrate postural control, muscular strength, balance, coordination, and gait into functional movement patterns representative of daily activities and school-related tasks.

Exercise Prescription

Exercise	Sets	Repetitions	Rest	Progression	Primary Target

Squats	3	12	45 s	Increase movement quality	Lower-limb strength
Step-ups	3	10 each leg	45 s	Increase step height	Functional strength
Walking with light load (≤10% body mass)	3	20 m	45 s	Increase walking efficiency	Gait stability
Dynamic plank variations	3	30 s	30 s	Increase exercise complexity	Core stability
Mountain climbers	3	20	45 s	Increase movement speed	Whole-body coordination
Controlled jumping drills	3	10	45 s	Increase landing control	Power & balance
Multidirectional running drills	3	20 m	45 s	Increase directional changes	Agility
Functional running games	3	60 s	60 s	Increase task complexity	Functional endurance

Phase Progression: Exercise progression was achieved by increasing movement complexity, coordination demands, speed of execution, and functional task integration while maintaining correct technique and age-appropriate intensity.

TRAINING PROGRAM REFERENCES

World Health Organization. (2020). *WHO Guidelines on Physical Activity and Sedentary Behaviour*. Geneva: World Health Organization.

Supports:

- At least 60 minutes of moderate-to-vigorous physical activity per day for children and adolescents.
- Physical activity recommendations for school-aged children.
- Structured physical activity programs aimed at improving health and physical fitness.

Faigenbaum AD, Kraemer WJ, Blimkie CJR, et al. (2009). *Youth Resistance Training: Updated Position Statement Paper From the National Strength and Conditioning Association. Journal of Strength and Conditioning Research*, 23(Suppl 5), S60–S79. https://doi.org/10.1519/JSC.0b013e31819df407

Supports:

- Body-weight resistance exercises.
- Core stabilization exercises (e.g., plank, bridge).
- Fundamental lower-limb strengthening exercises (e.g., squats).
- Progressive overload principles.
- Safe exercise prescription for children and adolescents.

Behm DG, Faigenbaum AD, Falk B, Klentrou P. (2008). *Canadian Society for Exercise Physiology Position Paper: Resistance Training in Children and Adolescents. Applied Physiology, Nutrition, and Metabolism*, 33(3), 547–561. https://doi.org/10.1139/H08-020

Supports:

- Safe resistance training practices in children and adolescents.
- Progressive exercise progression.
- Age-appropriate resistance training guidelines.
- Exercise safety and supervision.

Cox A, Fairclough SJ, Kosteli MC, Noonan RJ. (2020). *Efficacy of School-Based Interventions for Improving Muscular Fitness Outcomes in Adolescent Boys: A Systematic Review and Meta-analysis. Sports Medicine*, 50(3), 543–560. https://doi.org/10.1007/s40279-019-01215-5

Supports:

- School-based exercise interventions lasting 12–16 weeks.
- Development of muscular fitness in school-aged children.
- Structured intervention design.
- Evidence-based exercise programming in educational settings.

Wu J, et al. (2023). *School-Based Exercise Interventions: A Systematic Review and Network Meta-analysis. Frontiers in Public Health.* https://doi.org/10.3389/fpubh.2023.1194779

Supports:

- Balance training.
- Coordination exercises.
- Agility development.
- School-based physical activity interventions.

Zhu F, et al. (2025). *The Effect of School-Based Functional Training on Physical Fitness and Postural Health in Children. Scientific Reports.* https://doi.org/10.1038/s41598-025-31557-2

Supports:

- Functional training in children.
- Postural health improvement.
- Sixteen-week structured exercise interventions.
- School-based physical activity programs.
